# Trading and Shopping

**Published:** 1869-07

**Authors:** 


### TRADING AND SHOPPING.

BY E.

It may seem a singular subject to introduce to our American people who are noted the world over for the habit of trading. And yet, because of this very fact it is useful time and thought which is spent considering how to do it.

We are called a nation of *traders*. We were *traders* when in olden time we traded or gave up in exchange one kind of goods for another

There is but little of this business now done, we sell now more than we *trade*, i. e., we place a value in money upon some article which we wish to dispose of, and then seek for a person who will give us that price. I leave this view of trading to consider the matter of "ask and take," which is known as "shopping."

Webster defines shopping to be the "act of frequenting shops," and shops he says are "places where anything is *sold*," mind he does say *traded.*

Now we begin to understand one another. It is *shopping* that I am after. Those people who frequent our stores daily, looking about and asking to *see* goods, prefacing the request "to see" with the remark, "we do not intend to purchase, but we brought our friend, (or we came,) to see what pretty goods you have." This class is very numerous. Store after store employs two or three pleasant, willing and affable clerks to wait on these "lookers to see" what the store contains. As they have an influence with some other parties who *do* want to buy, they must be accommodated and treated kindly, lest they walk out of the doors, shaking not only the dust off their feet, but contempt and scowls from curled lips and indignant faces.

And now, there is another class, too large by far, who consider it a point of skill to try the patience and pride of the one who attends upon it. We Americans are boastful of our keenness and judgment. Immediately upon seeing a piece of goods, the customer of this class of which we are speaking, begins to criticise it. Too often we all forget to consider what is the meaning of the word critic. How many remember to "find out the merits of a thing considered"? How much oftener do we seek to find the faults of everything? Our customer immediately begins to seek for faults. In return, why should not the merchant endeavor to turn every attention to the beauties only. Thus, both sides are soon engaged in a deep game. This is not trading. It is a contest of wits, as concerns an article under consideration. The merchant has placed a value of dollars and cents on the wares—the customer does not exhibit in turn an article for exchange. He or she holds in hand a purse of money of already fixed value. Both know exactly how much that money is worth. It is not as though the customer offered an article for exchange which had been contrived or made by his or her own individual skill or patient labor. In this case it would be a proper and pleasing strife or competition of wit and brains. The wants of both would immediately determine the value.

If we return to the first conception of the transaction, the efforts of the merchant to present the beauties of his goods, as against the depreciating efforts of the buyer, we have an exhibition of more than half the daily labors of our stores.

But few customers say frankly to the merchant when they visit, "I am in search of such and such an article," naming as nearly as possible the thing, "and wish your judgment and taste to help me find it." I do not believe that an obliging customer, such as I describe, will ever be cheated knowingly by a *man* who cares for his good name as a man. The word implies too much worth and character to be sold for a petty bargain of a few dollars.

Let sharpers go and meet sharpers, but good customers seek good merchants, and very soon a look into a store by a stranger to it will be a sure guide as to whether the proprietor can be trusted or not.

Many a time I have seen a weary merchant after a patient humoring of the impertinent and ignorant criticism of his goods, turn at bay and offer some article at far less than its cost, to try whether the man or woman, whom he is showing, does know a bargain when offered. No, the customer don't know enough of what he or she asks for to recognize it even though lying under his or her hand, and disdains it because it is thought smart and wise to declare it was inferior to his or her appreciation. Poor fools both, the merchant to stand the imposition and the customer to stand on his wit.

The days are coming when a good merchant will not allow many of the remarks to be made of his wares which are made. Quietly and with dignity the goods will be returned to the shelf and his time be turned to a more appreciative patron. Then the grades of goods must be determined by their price and the word of both parties. The merchant's heart, if honest, will show in his actions, and the customer, who if ignorant, wishes advice, will find ready information, and goods no different than they are claimed to be, nor worse than expected. Of course a long story may be spun upon unjust and deceitful merchants. Remember my good listen er, for *you* are able to know what I mean, that the demand for honesty will create honesty. I know the ardent wish of most merchants is for honest clerks to sell honest goods to honest buyers. They who treat the merchant fairly, and with smiles brighten up many an hour of absolute disgust which comes every day to the shopman.

Don't try to know too much, nor try to exhibit your great intelligence. What you know will show itself worthily and be to your credit.

There are ladies who are known all through every place, for their good words and who will never be sold unfairly. Merchants remark one to another that they take pride in attending to Mrs. —— or Miss ——'s wants. "I don't always sell her," they say, "for she wanted what I could not claim for my goods. I sent her to you to get them." That woman could go from store to store and never find herself imposed upon. Ignorant perhaps as the "*shopper*" calls her, she gets her money's worth always, because she trusts and finds her weakness respected.

Really I could run around and around, always bringing up at the point I seek to lead you—make yourself known for a person who asks for what you wish and would rather be cheated once than to cheat yourself. You will always be known for just the value you place upon yourself. God's guinea stamp never wears off the face of the gold you are made of.

